# *Galleria mellonella* as an infection model for the multi-host pathogen *Streptococcus agalactiae* reflects hypervirulence of strains associated with human invasive disease

**DOI:** 10.1080/21505594.2019.1631660

**Published:** 2019-06-24

**Authors:** Anne Six, Sakranmanee Krajangwong, Margaret Crumlish, Ruth N. Zadoks, Daniel Walker

**Affiliations:** a Institute of Infection, Immunity and Inflammation, College of Medical, Veterinary and Life Sciences, University of Glasgow, Glasgow, UK; b Institute of Aquaculture, University of Stirling, Stirling, UK; c Institute of Biodiversity, Animal Health and Comparative Medicine, College of Medical, Veterinary and Life Sciences, University of Glasgow, Glasgow, UK

**Keywords:** Streptococcus agalactiae, Galleria mellonella, group B Streptococcus, infection model

## Abstract

*Streptococcus agalactiae*, or group B *Streptococcus* (GBS), infects diverse hosts including humans and economically important species such as cattle and fishes. In the context of human health, GBS is a major cause of neonatal infections and an emerging cause of invasive disease in adults and of foodborne disease in Southeast Asia. Here we show that GBS is able to establish a systemic infection in *Galleria mellonella* larvae that is associated with extensive bacterial replication and dose-dependent larval survival. This infection model is suitable for use with GBS isolates from both homeothermic and poikilothermic hosts. Hypervirulent sequence types (ST) associated with invasive human disease in neonates (ST17) or adults (ST283) show increased virulence in this model, indicating it may be useful in studying GBS virulence determinants, albeit with limitations for some host-specific virulence factors. In addition, we demonstrate that larval survival can be afforded by antibiotic treatment and so the model may also be useful in the development of novel anti-GBS strategies. The use of *G. mellonella* in GBS research has the potential to provide a low-cost infection model that could reduce the number of vertebrates used in the study of GBS infection.

## Introduction

*Streptococcus agalactiae*, or group B *Streptococcus* (GBS) is a Gram-positive multi-host pathogen and a major cause of human neonatal infections and mortality. GBS was initially described in 1887 as an animal pathogen responsible for mastitis in ruminants and although largely eradicated in some countries with a highly developed dairy industry, remains a major cause of bovine mastitis in many countries [1]. In 1966, GBS infection of fish was reported in the USA [2] with subsequent outbreaks described in fish farms in Asia and the Americas [3,4] and, most recently, in Africa [5]. GBS infection is often associated with high levels of mortality in fish and with the worldwide growth of aquaculture has a significant economic impact [6]. In the 1970s GBS was described as the leading cause of human neonatal infectious morbidity and mortality with two distinct GBS-associated syndromes: early-onset disease occurring 0–6 days after birth and late-onset disease occurring 7 days–3 months after birth [7,8]. Sequence type (ST) 17 is the most common strain affecting neonates and is recognized as a hypervirulent clone [9,10]. The implementation of routine antibiotic prophylaxis during labor in many countries has resulted in a decrease of the incidence of early-onset disease, but the number of cases of late-onset disease has remained stable or even increased in some countries [8,11]. In other countries, routine antibiotic prophylaxis is not recommended, in part because of concern about the potential impact on the infant microbiome [12]. GBS is also recognized as an emerging pathogen in adults that is associated with high fatality rates, reaching 15% in high-income countries [13–15]. Adult GBS infections are often associated with underlying co-morbidities, but a recent foodborne outbreak of invasive ST283 GBS disease in Singapore was linked to the consumption of raw fish and occurred in previously healthy adults [16,17]. Despite some level of host adaptation, e.g. association of ST17 with human neonatal infectious disease [10], ST67 with cattle [18] and association of ST7, ST283 and clonal complex (CC) 552 with poikilothermic animals [19], host-association is not absolute and interspecies transmission between people, cattle and fish may occur [16,20,21].

The routine use of antibiotic prophylaxis in pregnant women carrying GBS in many countries and routine use of antimicrobials in aquaculture and dairy farming may all exert selective pressure that favors the emergence of antimicrobial resistance (AMR) in GBS [22]. The first line of treatment against GBS infections in humans and cattle is beta-lactams antibiotics, such as penicillin, to which GBS strains were considered universally susceptible. Since 1994, cases of reduced penicillin susceptibility caused by mutations in Penicillin-Binding Protein 1a (PBP1a), PBP2b and PBP2x have been described in GBS from humans [23–25] and cattle [26]. In cases of penicillin allergy, macrolides and lincosamides, such as erythromycin and clindamycin, are considered to be the best alternative. Reported resistance to erythromycin ranges from just over 14% in South America [27] to around 30% in North America and Europe [28,29] and over 70% in China [30]. To limit the risk of disease and reduce reliance on antimicrobials for GBS control, vaccine development is an active area of research in humans [31,32] and fishes [6] and to a lesser extent in cattle [33]. Currently, there is no GBS vaccine commercially available for human or bovine use and commercially available fish vaccines do not provide cross-protection to all relevant serotypes. Consequently, the development of alternative anti-GBS strategies is of prime importance [34].

A number of *in vivo* models exists for GBS infection (mice, rats, zebrafish) and are valuable for the study of host–pathogen interactions and virulence [35–37]. In addition, challenge studies with GBS are conducted in the target host species, notably cattle and tilapia, rather than in model species [31,38]. To reduce the use of vertebrate animals in scientific research, larvae of the greater wax moth (*Galleria mellonella*) are increasingly being used as an alternate infection model for several human and non-human bacterial pathogens such as *Pseudomonas aeruginosa* [39], *Bacillus cereus* [40], *Listeria monocytogenes* [41,42], *Vibrio anguillarum* [43] and *Clostridium difficile* [44]. They have also been used to study the virulence of *Streptococcus* spp., notably *Streptococcus pyogenes* [45,46], *Streptococcus pneumonia* [47], and *Streptococcus suis* [48]. Their ease of use, short life span, and cost-effectiveness make them a useful tool for screening of new antimicrobials and pathogen mutant libraries [49,50]. The genome of *Galleria mellonella* has recently been sequenced [51] and transcriptome and microRNA data from larvae are available [52,53], further increasing the appeal of the model. While the larvae rely entirely on the innate immune system and do not possess an adaptive immune system, their innate immune system has remarkable similarities in terms of function to the innate immune system of vertebrates [49,54]. Indeed, phagocytosis in insects and mammals is believed to be similar, with the hemolymph of larvae containing phagocytic immune cells called hemocytes similar to neutrophils found in blood. Additionally, bacterial infection results in the production of antimicrobial peptides (AMPs) by the insect fat body, the equivalent of the mammalian liver, and an increase of reactive oxygen species generation [55–57].

Here, we evaluate the use of *Galleria mellonella* larvae as an *in vivo* model of infection for GBS by testing their susceptibility to human, bovine or fish GBS isolates encompassing host-associated and multi-host STs. We also determine the utility of *G. mellonella* larvae for *in vivo* screening of antimicrobial efficacy during GBS infection to explore its potential use in the evaluation of new antibacterial treatments.

## Results

### *G. mellonella* larvae are susceptible to GBS infection

**Figure 1.**
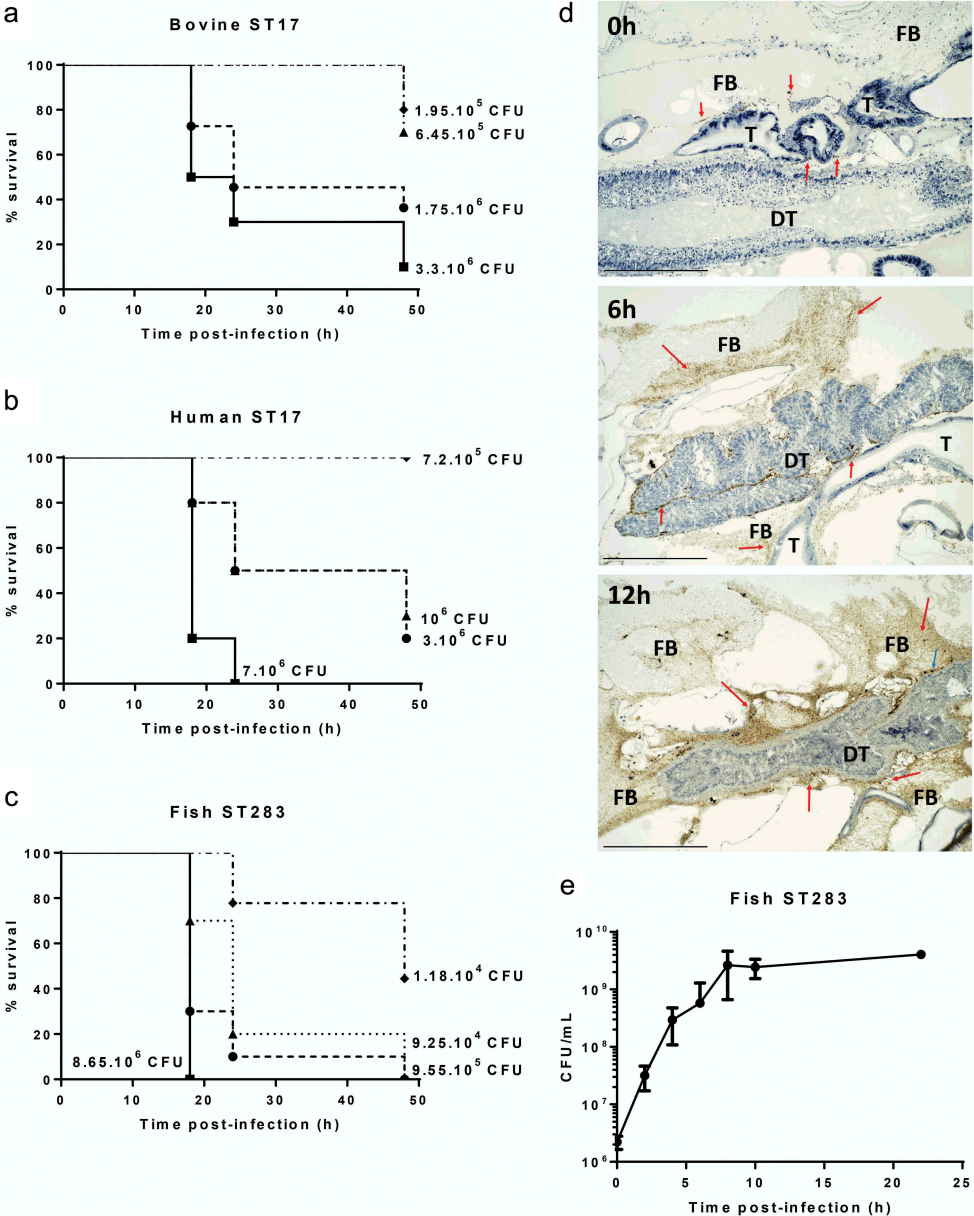
Virulence and replication of group B *Streptococcus* (GBS) in the *Galleria mellonella* larvae model. Kaplan–Meier survival curves of larvae challenged with serial dilutions of ST17 bovine isolate MRI Z1-363 (A), ST17 human isolate MRI Z2-093 (B), ST283 fish isolate STIR-CD-25 (C). All survival curves show one representative experiment, with use of 10 larvae/group. PBS-injected larvae were used as a negative control, and all survived until the endpoint of the experiment. (D) Immunohistochemical analysis shows extensive GBS replication in *G. melonella*. Larvae were inoculated with 10^6^ CFU of MRI Z2-366 (ST283) and collected immediately after inoculation and 6 h and 12 h postchallenge. GBS was detected through immunohistochemical staining of sections. For histological examination, sections were stained with hematoxylin and eosin. Scale bar 500 µM. Abbreviations: DT, digestive tract; FB, fat body; T, trachea. Red arrows indicate the presence of GBS, visible based on brown staining, and blue arrows indicate phagocytic cells. (E) Bacterial counts of homogenized larvae, from two distinct experiments using 3 larvae per time point, showing *in vivo* growth of GBS ST283 isolate STIR-CD-25 in *G. mellonella* larvae following infection with 10^6^ CFUs. Errors bars represent the standard deviation (SD).

To begin to assess the suitability of *G. mellonella* larvae as an infection model for GBS isolated from its major hosts, we tested the susceptibility of larvae to dose-dependent killing by GBS from human, bovine and piscine origin. Larvae were challenged with serial dilutions of two STs that are hypervirulent in humans, i.e. ST17 and ST283, represented by isolates of ST17 (human MRI Z2-093 and bovine MRI Z1-363) and one isolate of ST283 (piscine STIR-CD-25). Infected larvae were incubated at 37°C and survival were monitored at 18 h, 24 h, and 48 h postinfection (representative example in Figure 1(A-C), additional replicates in Figure S1(A-C)). All isolates induced a dose-dependent response that was reproducible for each isolate in three independent experiments. Immunohistochemical analysis of infected larvae with an anti-GBS antibody indicated extensive bacterial replication within larvae during the course of infection, particularly in the fat body and the tissue surrounding the digestive tract (Figure 1(D)). To generate quantitative data on the extent of GBS replication, CFU counts from homogenized infected larvae were measured for the piscine ST283 isolate STIR-CD-25 (Figure 1(E)). CFU counts show extensive growth of ST283 up to 8 h postinfection, which is followed by a stationary phase.

**Figure 2.**
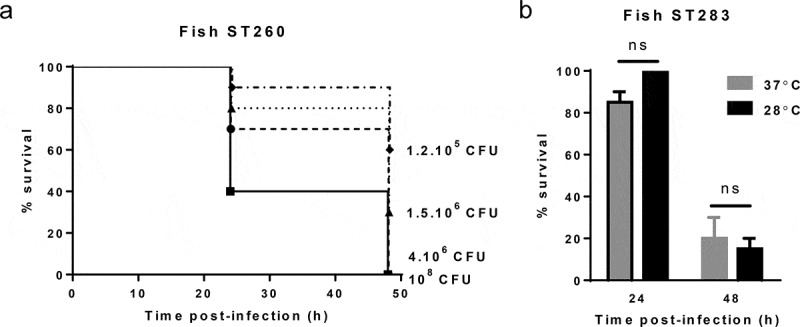
Virulence of group B *Streptococcus* (GBS) in the *Galleria mellonella* infection model is largely temperature independent. (A) Kaplan–Meier survival curves of larvae challenged with serial dilutions of ST-260 fish isolate STIR-CD-10 demonstrating dose-dependent survival at 28°C. Survival curves show one representative experiment, with use of 10 larvae per group. PBS-injected larvae were used as a negative control, and all survived until the endpoint of the experiment. (B) Survival of larvae at 24 h and 48 h postinfection by ST283 fish isolate (MRI Z2-366, 9.10^5^ CFU/mL) with incubation at 37°C or 28°C. Data were collected from two distinct experiments with 10 larvae per group for each experiment, errors bars represent the SD (ns: not significant; unpaired t-test).

To further asses the utility of this model across a range of GBS strains, we tested the susceptibility of larvae to the fish-specific ST260 isolate STIR-CD-10. Unlike piscine isolates of ST7 or ST283, which belong to clonal complexes that also affect humans, ST260 belongs to clonal complex 552, which is only found in cold-blooded animals, and grows optimally at 28°C. Similar to experiments with non-ST260 strains at 37°C, ST260 (piscine isolate STIR-CD-10) displayed dose-dependent killing of *G. mellonella* larvae at 28°C (Figure 2(A), Figure S1(D)). To determine if incubation temperature is a critical parameter in determining the course of infection as has been previously described for a *Galleria* model of *Streptococcus pyogenes* [45], one ST283 isolate (piscine MRI Z2-366) was used to challenge larvae at both 28°C and 37°C. Percentage survival 24 h and 48 h postinfection was similar at both temperatures, suggesting that the temperature of incubation post-challenge does not greatly influence virulence of GBS in this model (Figure 2(B)).

### Strains associated with hypervirulence in humans show increased virulence in *G. melonella*

**Figure 3.**
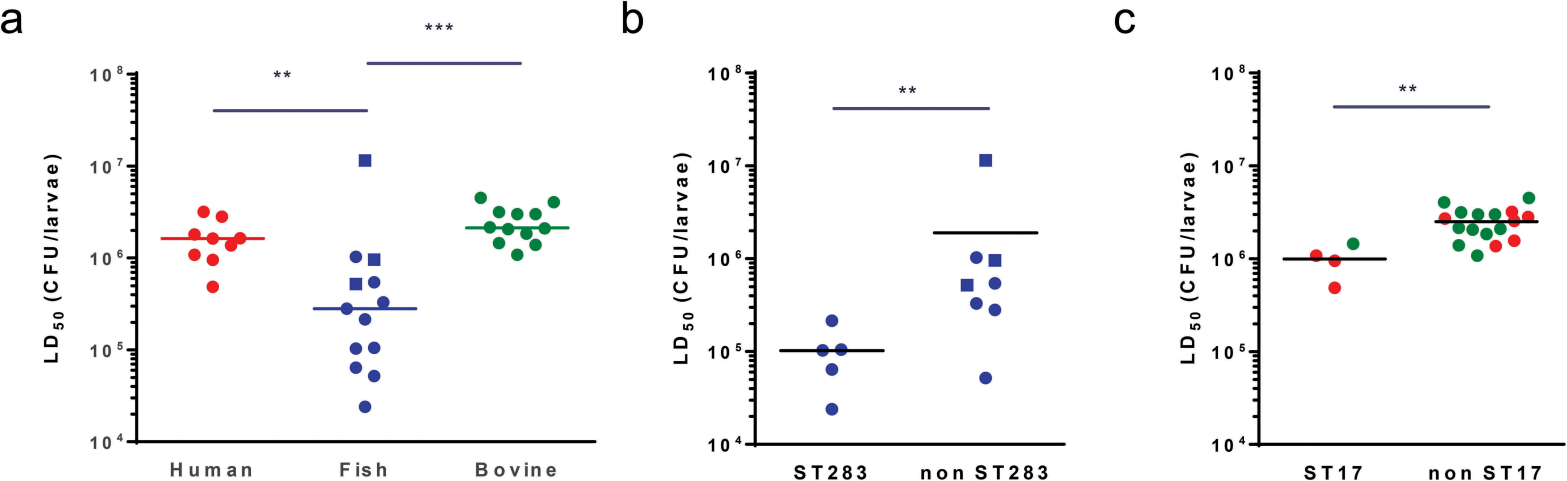
Group B *Streptococcus* (GBS) strains associated with hypervirulence in humans show increased virulence in *Galleria mellonella*. LD_50_ values determined by Probit analysis following infection of larvae by (A) *Streptococcus agalactiae* human (red), fish (blue) and bovine (green) isolates at 28°C (ST260 fish isolates, square symbols) or 37°C (non-ST260 isolates, round symbols), by (B) fish ST283 and non-ST283 isolates and by (C) human (red) and bovine (green) ST17 and non-ST17 isolates. Each datapoint represents the mean LD50 of at least two experiments in which groups of 10 larvae were infected with 4 different inoculums, errors bars represent the SD (**: *p* < 0.01; ***: *p* < 0.001; Mann–Whitney test).

Next, we examined if isolates from different host species showed different levels of virulence in *G. melonella* larvae through the determination of the LD_50_ for GBS isolates of human (n = 9), bovine (n = 12) and piscine origin (n = 13). For each host species, we selected isolates representing major epidemiologically relevant capsular serotypes and clonal complexes, including those that are found across multiple host species (**Table S1**). LD_50_ values for all isolates following infection of larvae at 37°C are shown in Table S1. Overall, LD_50_ values of human and bovine isolates were relatively homogenous with values ranging from 4.9 × 10^5^ to 3.2 × 10^6^ CFU and 1.1 × 10^6^ to 4.5 × 10^6^ CFU, respectively. Interestingly, LD_50_ values obtained for infection with fish isolates at 37°C showed a wider range (from 2.4 × 10^4^ to 1.1 × 10^7^ CFU) and were significantly lower than those of human and bovine isolates, indicating that fish isolates are more virulent in the *Galleria* model (Figure 3(A)). This apparent host-effect was largely driven by ST283, as ST283 isolates were more virulent than non-ST283 isolates from fish (Figure 3(B)). Within the non-piscine isolates, ST17 isolates showed the highest virulence, with the comparison of LD50 between ST17 and non-ST17 groups showing a small but significant difference (Figure 3(C)). There was no significant correlation between capsular serotype and virulence (Figure S2).

### Antibiotic treatment rescues larvae infected with susceptible strains

**Figure 4.**
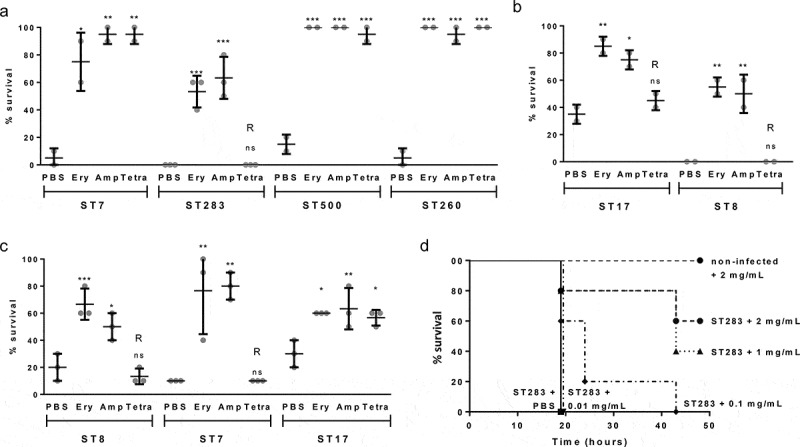
Antibiotic treatment affords survival of *Galleria mellonella* larvae against a lethal dose of group B *Streptococcus* (GBS). (A-C) Survival of treated (injection of 10 µL of 2 mg/mL erythromycin, ampicillin or tetracycline 2 hours post-infection) and untreated (10 µL of sterile PBS) larvae 48 hours post infection with GBS fish (A), human (B) or bovine (C) isolates. R indicates that the isolate is resistant to the corresponding antibiotic. Data was collected from at least 2 distinct experiments with 10 larvae per group for each experiment and error bars represent the SD (ns: non significant, *: p < 0.05, **: p < 0.01; ***: p < 0.001; one-way ANOVA). (D) Kaplan-Meier survival curves of larvae challenged with 9.10^6^ CFUs of ST-283 fish isolate STIR-CD-25 demonstrating dose-dependent efficacy of ampicillin treatment. Survival curves show one representative experiment, with 5 larvae per group. Groups of non-infected larvae received injections of PBS or ampicillin (2mg/mL, 1mg/mL, 0.1mg/mL or 0.01mg/mL) were used as a negative control, and all survived until the endpoint of the experiment. Only the control group injected with 2mg/mL ampicillin is shown on the graph.

To test the utility of the *Galleria* model to screen for antibiotic efficacy against GBS infection *in vivo* we tested the ability of erythromycin, ampicillin, and tetracycline to afford survival against a lethal GBS infection. Because of the wide range of LD50 values observed for fish isolates, one representative for each ST from fish isolates (ST7, ST283, ST500, ST260) was included. For human isolates, a hypervirulent and a non-hypervirulent ST were selected (ST17, ST8), whilst bovine isolates were selected to match STs from fish- or human-derived isolates if available (ST8, ST7, and ST17). One ST260 fish isolate (STIR-CD-10) was tested by challenging the larvae and incubating at 28°C post-infection with the other isolates tested at 37°C. Prior to infection studies, the MIC of each isolate to erythromycin, ampicillin, and tetracycline was determined by the broth dilution method (Table S1). All isolates tested are susceptible to erythromycin and ampicillin with MICs ranging from 0.031 to 0.5 µg/ml. One fish (ST283; STIR-CD-25), two human (ST17; MRI Z2-093 and ST8; MRI Z2-137), and two bovine (ST8; MRI Z2-053 and ST7; MRI Z1-354) isolates were found to be resistant to tetracycline (MIC ranging from 16 to 64 µg/ml) while the other tested isolates are susceptible (MICs ranging from 0.125 to 1 µg/ml). Except for those isolates identified as resistant to tetracycline, one injection of 20 µg of erythromycin, ampicillin or tetracycline 2 hpost infection, was able to rescue larvae infected by fish, human or bovine isolates (Figure 4(A-C)). For those isolates resistant to tetracycline survival of *Galleria* was not significantly different on tetracycline treatment from control larvae treated with PBS after challenge. These data indicate an excellent correlation between GBS sensitivity to antibiotics *in vitro* and *in vivo* at both 37°C and 28°C, indicating this model may be useful for testing novel anti-GBS agents. To further characterize the use of the Galleria infection model for drug-screening assay, groups of larvae infected with an ST283 fish isolate were treated with increasing concentration of ampicillin, showing that survival increase in a dose-dependent manner (Figure 4(D), additional replicate in Figure S3).

## Discussion

In recent years, the use of *Galleria mellonella* larvae as bacterial infection model has been developed as an alternative to murine or other vertebrate models of infection to contribute to the 3Rs (reduction, replacement, and refinement) of animal use in scientific research. In this work, we demonstrate the utility of *G. mellonella* larvae as a GBS infection model. Unlike previously evaluated *Streptococcus* species, which are predominantly associated with a single host species, GBS is a multi-host pathogen, and we provide, to our knowledge, the first comparison of virulence in this model for *Streptococcus* isolates obtained from clinical disease across multiple host species. GBS disease isolates are able to establish systemic infection of *G. mellonella* larvae with extensive bacterial replication and dose-dependent larval survival. The model is broadly applicable, with GBS isolates from multiple hosts, encompassing both homeothermic and poikilothermic species, able to establish a lethal infection. Unlike other invertebrate models such as *Caenorhabditis elegans* and *Drosophila melanogaster, G. mellonella* larvae are able to survive at 37°C and 28°C, enabling the study of GBS infection in the range of relevant host-specific temperatures [58].

Other *in vivo* models of GBS infection exist, including models that have been used for comparison of isolates from different host species, e.g. human and bovine isolates in a bovine mammary gland challenge or human, bovine and piscine isolates in a tilapia challenge model [31,59]. Major drawbacks of such models are the fact that they can only be used at a temperature that is appropriate for the homeothermic or poikilothermic host species used, and that they fail to reduce the use of vertebrate animals in research. Use of *Galleria* overcomes both limitations, although the model has limitations of its own. For example, GBS virulence in bovines is associated with the presence of the lactose metabolism operon Lac.2, which is largely absent from non-bovine isolates. The utilization of lactose (milk sugar) by GBS bovine strains facilitates bacterial survival and infection in milk-producing mammary glands but would not confer a survival advantage in other tissues [60,61]. This is reflected in lack of a significant difference in virulence of human and bovine isolates in our model. Conversely, a mobile genetic element that encodes galactose metabolism is thought to be associated with GBS virulence in fishes, regardless of ST, yet the *Galleria* model shows that ST283 is more virulent than other fish-associated strains [38]. Recently, the use of a clinical ST283 human isolate in a murine model showed a clear increase in virulence compared to that of a clinical ST23 human isolate [62], but the basis for the hypervirulence of this ST remains largely unknown. The hypervirulence of ST283 strains in the *Galleria* model raises the possibility of utilizing this cost-effective model to screen transposon or other mutant libraries to identify key GBS virulence factors specific to this sequence type. Likewise, the hyper-virulent ST17 isolates showed a slight increase in virulence compared to homeothermic non-ST17 strains, suggesting that at least some specific virulence factors are reflected in the *G. mellonella* larvae model. Testing the virulence of deletion mutants of known ST17-specific virulence factors, such as HvgA, would provide more information on the model’s utility for study of mechanism contributing to GBS virulence in humans.

Due to the importance of GBS as a human, livestock and aquaculture pathogen and the widespread use of antibiotics to prevent or treat GBS infections, there is a pressing need to identify and develop new anti-GBS strategies that could limit the associated spread of antimicrobial resistance. In this respect, invertebrate infection models are very attractive for the screening of new antimicrobials, to compare their efficacy to known antibiotics, or study the effect of antibiotic combinations [35,49,63,64]. To demonstrate the potential to use *G. mellonella* larvae for *in vivo* screening of antimicrobials we show that known antibiotics are able to afford protection against a lethal GBS infection. Indeed, the systemic injection of commonly used anti-GBS antibiotics, such as erythromycin, ampicillin or tetracycline, in the hemolymph of infected larvae is able to rescue those infected with susceptible strains but not those infected with resistant strains. This model could, therefore, be used as a bridge between *in vitro* studies and the more relevant and costly animal models, such as rodents for human isolates or cattle for bovine isolates, to test the efficacy of novel antimicrobials compounds. However, while the *G. mellonella* larvae model is useful to test the efficacy of antimicrobials compounds such as antibiotics, the lack of adaptive immune system renders it useless for the screening of potential vaccines.

Overall, we show that the *G. mellonella* larvae model can be used as an infection model for GBS isolates from all host species and reflects the hypervirulence of ST283 and ST17, both of which are associated with invasive disease in humans. Furthermore, we show that this model can be used as a bridge between *in vitro* and *in vivo* studies for the screening of antimicrobial compounds.

## Materials and methods

### Bacteria and growth conditions

GBS isolates used in this study are described in Table S1. The panel of isolates represents a range of capsular serotypes and STs found in the three major host categories (human, bovine and fish). All human isolates originated from urinary tract infections so that potential differences in virulence between isolates within a host species could be associated with ST or serotype rather than clinical origin. Similarly, all bovine isolates originated from the milk of animals with mastitis. Where possible, human and bovine isolates of each ST were included to allow for assessment of host effects within ST. For fish isolates, the range of available STs was limited and represented the major CCs and serotypes known to be associated with naturally occurring infection in fishes (ST7-serotype Ia; ST283-serotype III; CC552-serotype Ib) [19]. GBS was stored at −80°C in 25% (v/v) glycerol and cultured in Brain Heart Infusion broth/agar medium (BHI, Oxoid) at 37°C, with the exception of ST260, a member of the poikilothermic CC552, which were grown at their optimal temperature of growth, 28°C with the media and conditions used.

### Galleria mellonella challenge

*G. mellonella* larvae were obtained from Livefood UK. They were kept in darkness at room temperature and were used maximum one week following arrival. Healthy larvae measuring from 2 to 2.5 cm in length and showing no signs of melanization were used for all experiments. GBS isolates were grown in BHI at 37°C or 28°C to an OD_600_ = 0.4–0.6. Cells were then washed twice in sterile PBS and diluted to the desired inoculum in PBS. Inoculums were serially diluted and plated on BHI agar plates just before administration for CFU counting. For the determination of the LD_50_ of GBS isolates, 4 groups of 10 larvae were injected with 10 µL of serial dilutions of bacterial suspension in the hemocoel via the last right pro-limb. Following challenge, larvae were placed in an incubator at 37°C or at 28°C in the case of ST260 isolates. Survival was followed for 48 h, larvae were considered dead when non-responsive to touch. LD_50_ was calculated using the Probit method (XLSTAT software). Survival curves were plotted using the Kaplan–Meier method and differences in survival were calculated using the log-rank test (GraphPad Prism 6). Experiments were done at least twice. One representative experiment is shown in the main text for survival curves, with additional data shown in the Supplementary material. Differences in LD_50_ between strains defined based on host, ST or serotype were assessed using Mann–Whitney test. To assess antibiotic efficiency, larvae were challenged with the LD_90_ of the GBS isolate tested and injected 2 h postinfection with 10 µL of 2 mg/mL erythromycin (Duchefa Biochemie), ampicillin (Melford Biolaboratories Ltd) or tetracycline (Sigma) in the hemocoel via the last left pro-limb. For all challenge experiments, a sham-inoculation of PBS was used for uninfected control larvae (n = 10 per experiment unless stated otherwise) to account for mortality caused by physical injury or infection by a contaminant. All larvae inoculated with PBS survived. Differences in survival rates between treated and untreated larvae were assessed using one-way ANOVA test followed by Dunnett test.

To assess the dose–response of ampicillin, groups of 5 larvae were challenged with a lethal dose of ST283 fish isolate STIR-CD-25 and injected 2 hpost infection with 10 µL of ampicillin at 2.0, 1.0, 0.10 or 0.010 mg/mL. Experiments were performed 3 times and sham-inoculation of PBS or ampicillin was used for uninfected control larvae. Dose-dependent survival was observed with increasing ampicillin concentration (Figure 4(D), with replicate shown in Figure S3).

### In vivo GBS growth curve

For the *in vivo* growth curve experiment, larvae were challenged with 10^6^ CFU of GBS. At each time point, groups of 3 larvae were kept at −20°C for 10 min before being transferred to Eppendorfs containing 100 µL of sterile PBS, homogenized, serially diluted and plated on chromogenic selective GBS Brilliance agar for quantification (Thermo Scientific, Basingstoke UK; PO5320A). Homogenization was performed using FastPrep lysing matrix M (MPbio).

### Immunohistochemistry (IHC) and histological analysis

Larvae were challenged with 10^6^ CFU of a fish-derived ST283 isolate (MRI Z2-366). Uninfected and infected larvae were collected immediately after inoculation, and 6 h and 12 h postchallenge and kept at −20°C for up to 10 min before fixation. Whole larvae were placed in 10⨰ volume of 10% neutral buffer formalin at room temperature for 48 h. Longitudinal and cross sections of paraffin-embedded larvae were prepared to detect GBS presence in an early, middle and late phase of challenge. For antigen retrieval, heat-induced epitope retrieval (HIER) was carried out using a Menarini Access Retrieval Unit (Biocare LLC, California, USA), in 10 mM Sodium Citrate buffer, pH 6.0 for 1 min 40 sat 125°C full pressure. Slides were loaded on to Dako Autostainer (Dako Colorado, INC., Colorado, USA) and rinsed 5 min with a Tris-buffered saline solution (TBS), pH 7.6 containing 0.05% Tween 20. Endogenous peroxidase was blocked with Dako REAL™ Peroxidase-Blocking Solution (S2023) for 5 min and then rinsed with buffer. Slides were incubated 30 min at room temperature with anti-Streptococcus group B antibody ab53584 (Abcam, Cambridge, UK) diluted 1: 200 in Dako universal diluent (S2022), washed, and then further incubated with anti-rabbit horseradish-peroxidase-labeled polymer (Dako, K5007ENV) for 30 min at room temperature. After incubation with 3,3ʹ-diaminobenzidine (5 min, RT; Dako, K5007 DAB), the slides were rinsed three times with hydrogen peroxide and counterstained with Gills Haematoxylin for 27 s. Pictures were acquired on EVOS Fl Auto 2 Imaging System (Invitrogen).

### Antibiotic*s*

The minimum inhibitory concentration (MIC) was evaluated using the broth dilution technique for strains used in the assessment of antibiotic efficiency. Microdilution plates containing 200 µL BHI broth medium (Oxoid) with decreasing concentration of antibiotics (erythromycin, Duchefa Biochemie; ampicillin, Melford Biolaboratories Ltd; tetracycline, Sigma; 256–0.125 µg/mL) were inoculated from overnight cultures. After overnight incubation, cultures were checked for growth, with the MIC being the lowest concentration of antibiotics that prevents visible growth.
