# Supplementary material for: Galleria mellonella as an infection model for the multi-host pathogen Streptococcus agalactiae reflects hypervirulence of strains associated with human invasive disease
Source: Virulence. 2019 Jun 24;10(1):600–9. doi: 10.1080/21505594.2019.1631660 (PMC6592362; doi:10.1080/21505594.2019.1631660)
Supplement: Supplemental Material [file kvir-10-01-1631660-s001.docx]

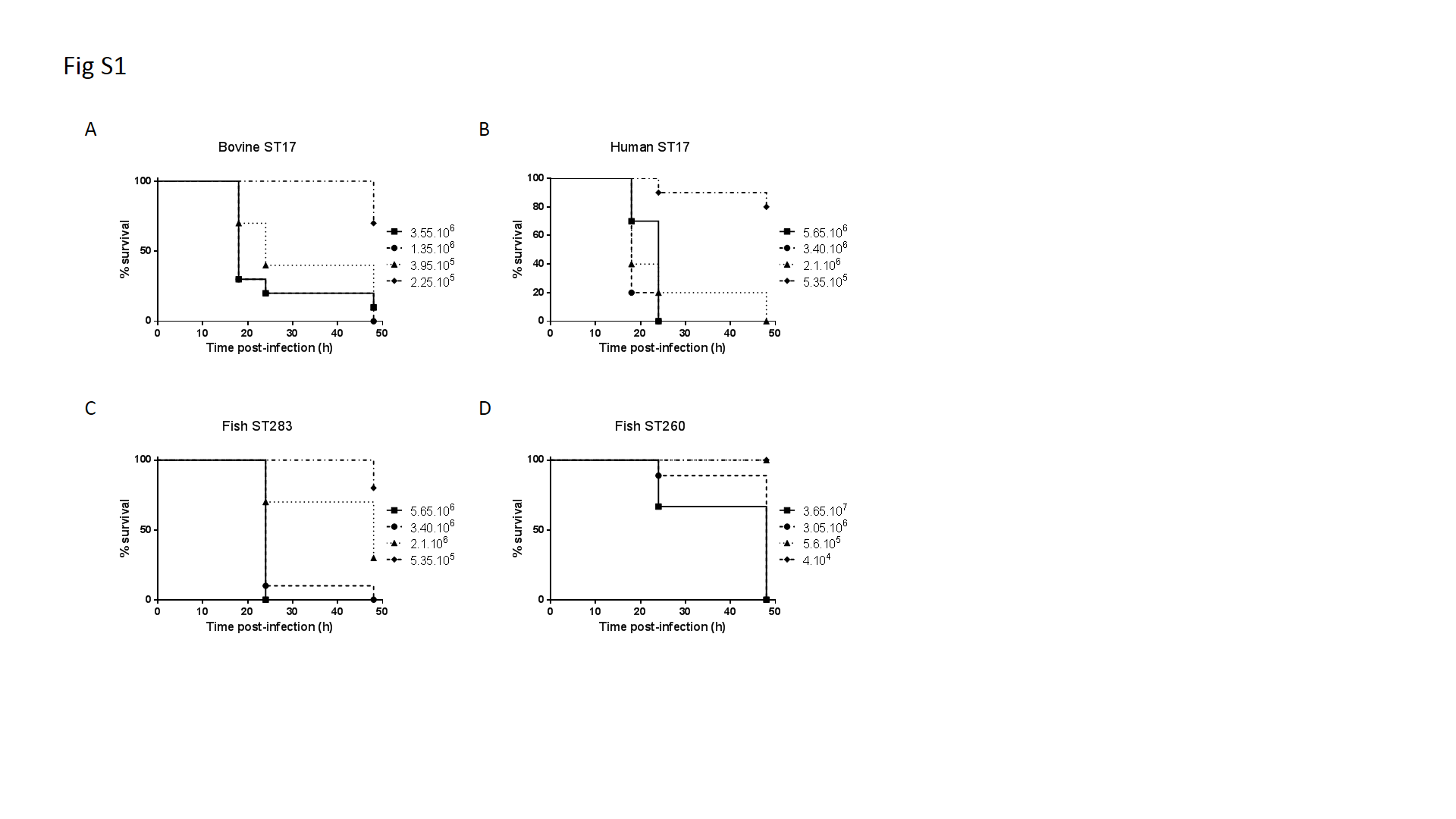


**Figure S1. Virulence of group B *Streptococcus* (GBS) in the *Galleria mellonella* larvae model.** Kaplan-Meier survival curves of larvae challenged with serial dilutions of ST17 bovine isolate MRI Z1-363 (**A**), ST17 human isolate MRI Z2-093 (**B**), ST283 fish isolate STIR-CD-25 (**C**) and ST260 fish isolate STIR-CD-10. All survival curves show one representative experiment, with use of 10 larvae / group. PBS-injected larvae were used as a negative control, and all survived until the endpoint of the experiment


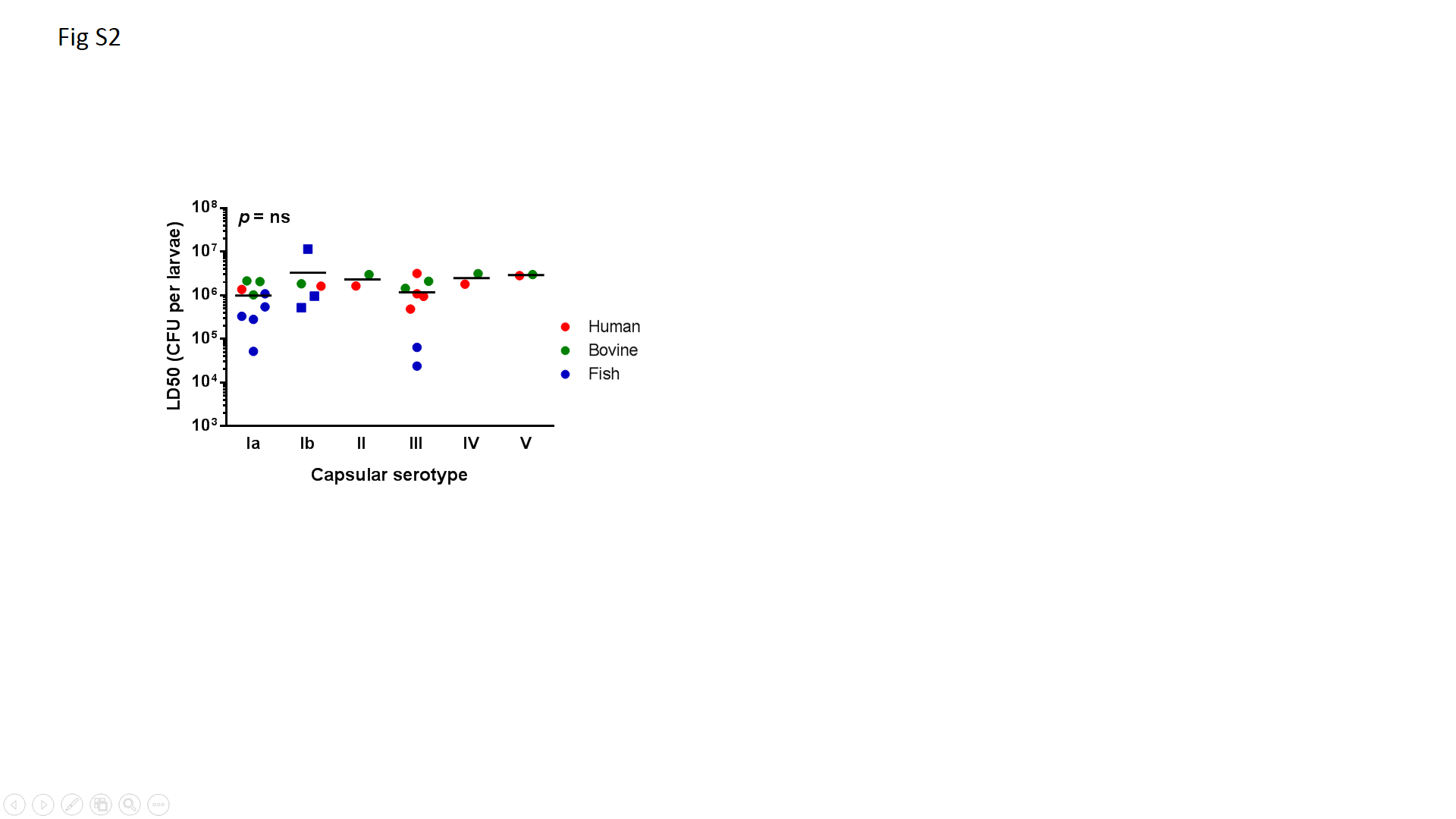


**Figure S2. Virulence of group B *Streptococcus* (GBS) isolates is not dependent on capsular serotype in the *Galleria mellonella* larvae model.** LD_50_ values determined by Probit analysis following infection of larvae by *Streptococcus agalactiae* human (red), bovine (green) and fish (blue) isolates at organised by capsular serotype. Each data point represent the mean LD50 of at least two experiments in which groups of 10 larvae were infected with 4 different inoculums (ns : non significant, Kruskal-Wallis test).


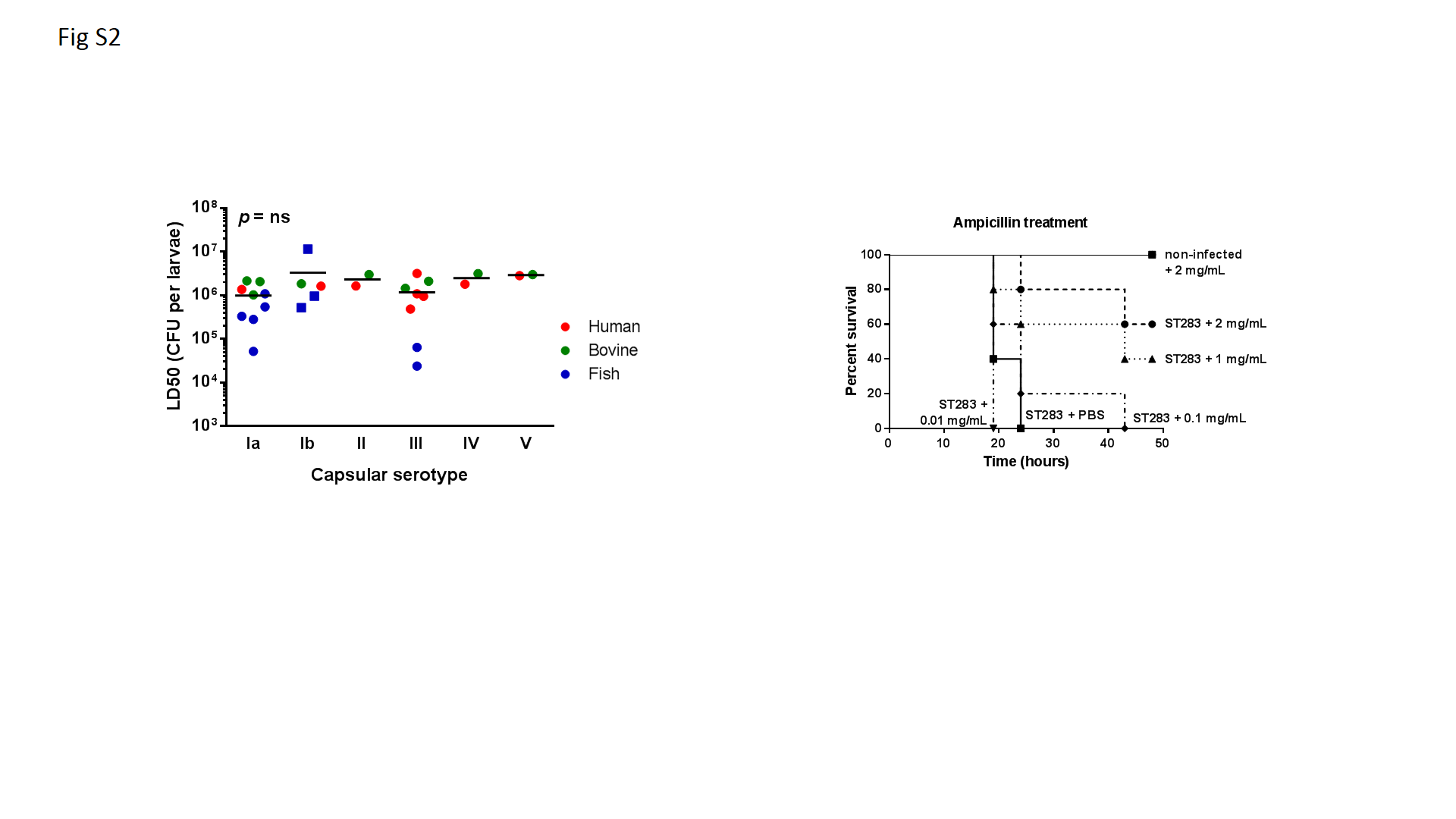


**Figure S3. Antibiotic treatment affords survival of *Galleria mellonella* larvae against a lethal dose of group B *Streptococcus* (GBS).** Kaplan-Meier survival curves of larvae challenged with 5.10^6^ CFUs of ST-283 fish isolate STIR-CD-25 demonstrating dose dependent efficacy of ampicillin treatment. Survival curves show one representative experiment, with use of 5 larvae per group. Groups of non-infected larvae received injections of PBS or ampicillin (2mg/mL, 1mg/mL, 0.1mg/mL or 0.01mg/mL) were used as a negative control, and all survived until the endpoint of the experiment. Only the control group injected with 2mg/mL ampicillin is shown on the graph.
